# Supplementary material for: Anti-Biofilm Activity of Oleacein and Oleocanthal from Extra-Virgin Olive Oil toward Pseudomonas aeruginosa
Source: Int J Mol Sci. 2024 May 6;25(9):5051. doi: 10.3390/ijms25095051 (PMC11084407; doi:10.3390/ijms25095051)
Supplement: Supplementary file 1 [file ijms-25-05051-s001.zip › SupplementaryTable S1.pdf]

Supplementary Table S1. Antibiotic susceptibility of human clinical isolates of *P. aeruginosa*

P. aeruginosa strains	Antibiotic class	Antibiotic agent	MIC (µg/ml)	Breakpoint (S/R)
**PA BL**				
	Cephalosporins	Ceftazidime	16	R
		Cefepime	16	R
	Beta lactam inhibitor combinations	Ceftazidime/avibactam	≥16	R
		Ceftolozane/ tazobactam	2	S
		Piperacillin/tazobactam	≥128	R
	Fluoroquinolones	Ciprofloxacin	≥4	R
	Polymixin	Colistin	≤0.5	S
	Aminoglycosides	Gentamicin	≥16	IE
		Tobramycin	≥16	R
		Amikacin	32	R
	Carbapenems	Imipenem	≥16	R
		Meropenem	≥16	R
**PA SW**	Cephalosporins	Cefepime	2	S
		Ceftazidime	2	I
	Beta lactam inhibitor combination	Ceftazidime/avibactam	2	S
		Ceftolozane/ tazobactam	0.5	S
		Piperacillin/tazobactam	≤4	I
	Fluoroquinolones	Ciprofloxacin	0.5	I
	Aminoglycosides	Gentamicin	≤1	IE
		Tobramycin	≤1	S
		Amikacin	2	S
	Carbapenems	Imipenem	1	I
		Meropenem	≤0.25	S

S = susceptible; R = resistant; IE = insufficient evidence; I = increased exposure. 1 Breakpoints established by EUCAST (EUCAST guideline version 12.0, January 2022).
