# Supplementary figures and images for: Anti-Biofilm Activity of Oleacein and Oleocanthal from Extra-Virgin Olive Oil toward Pseudomonas aeruginosa
Source: Int J Mol Sci. 2024 May 6;25(9):5051. doi: 10.3390/ijms25095051 (PMC11084407; doi:10.3390/ijms25095051)

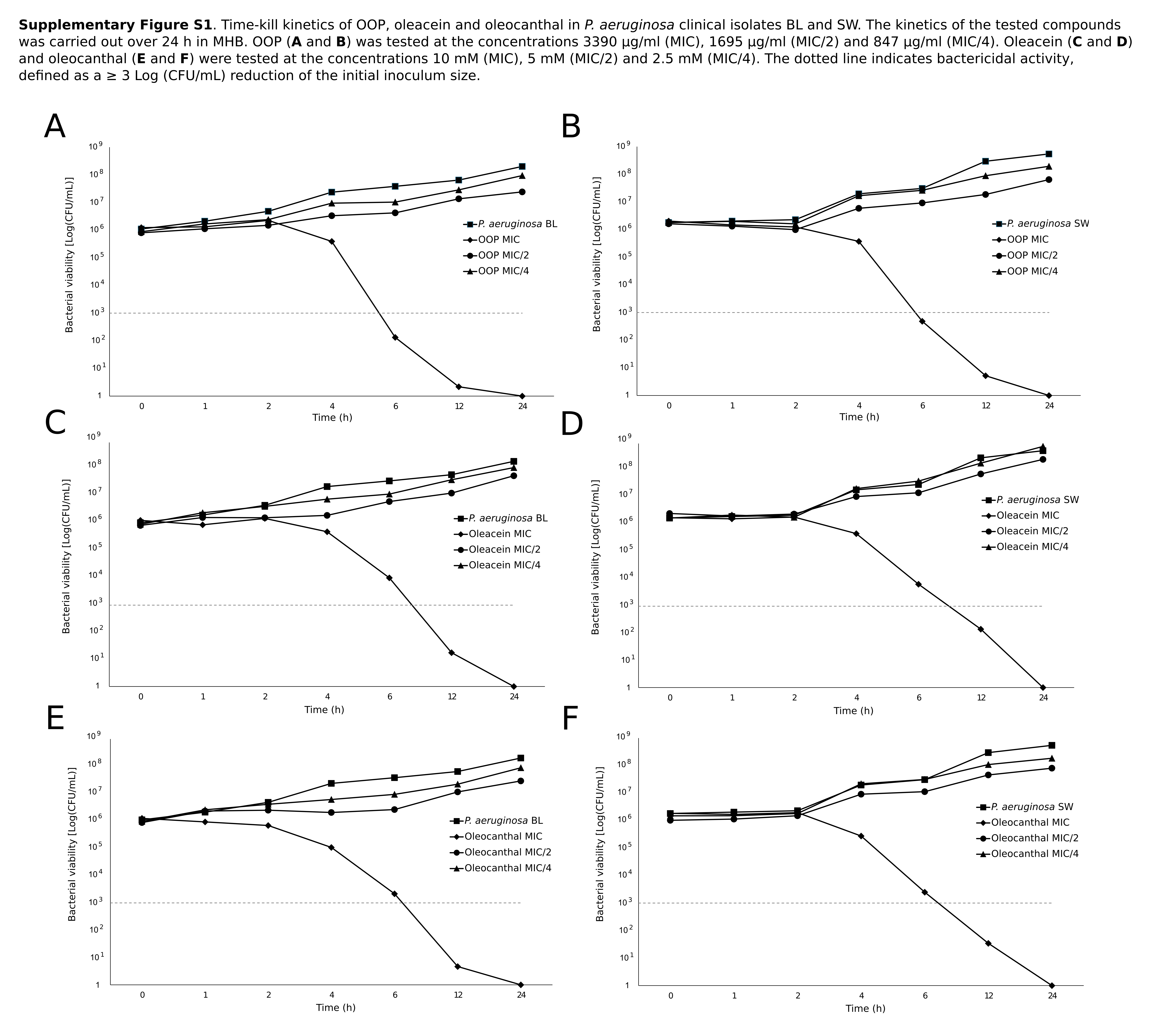

Supplement: Supplementary file 1 [file ijms-25-05051-s001.zip › SupplementaryFigureS1.tif]
